# Repair what is lost: Neuroprotection through neural stem cells in progressive MS

**DOI:** 10.1016/j.xcrm.2023.100985

**Published:** 2023-03-21

**Authors:** Menno M. Schoonheim, Eva M.M. Strijbis

**Affiliations:** 1 MS Center Amsterdam, Anatomy and Neurosciences, Vrije Universiteit Amsterdam, Amsterdam Neuroscience, Amsterdam UMC location VUmc, Amsterdam, the Netherlands; 2 MS Center Amsterdam, Neurology, Vrije Universiteit Amsterdam, Amsterdam Neuroscience, Amsterdam UMC location VUmc, Amsterdam, the Netherlands

## Abstract

Genchi et al.^1^ report the first phase 1 trial of neural stem cell transplantation in multiple sclerosis showing a reduction in gray matter atrophy. Results give hope for a new era of induced neuroprotection, especially in progressive multiple sclerosis.

## Main text

Multiple sclerosis (MS) is a disease of the central nervous system (CNS) driven by an auto-immune response to myelin. The pathological mechanisms that drive the multifocal inflammation so characteristic of MS are still unknown.^2^ Most people with MS feature periods of relapses, associated with the formation of new lesions, and periods of remission. Treatment of this relapsing-remitting phenotype is focused on preventing relapses with immunomodulating and immunosuppressive medications or hematopoietic stem cell transplantation (HSCT).^3^ However, there are important pathological changes outside these bouts of focal inflammation, and an increasing focus lies on mechanisms that drive slow neurological decline.^4^ Important among these is neurodegeneration,^5^ which strongly correlates with clinical functioning and can be severe in the form of brain atrophy but remains poorly understood.^2^ Such changes are especially severe in progressive MS (PMS), a phenotype associated with less relapses and a more gradual and more severe decline in clinical functioning. Immune-focused treatments have mostly been ineffective in PMS, highlighting the need for treatments focused on neuroprotection.

In a recent issue of *Nature Medicine*, Genchi et al.^1^ show the feasibility of neural stem cell transplantation in the STEMS trial. This is the first in-human phase 1 trial with intrathecally injected neuronal precursor cells (NPCs) from human fetal CNS (hfNPCs). The trial was based on the hypothesis that NPCs can exert trophic support and immunomodulation, leading to neuroprotection and tissue repair. As such, this is a significantly different approach from the anti-inflammatory compounds that have been trialed in PMS. The study is also important in that it features a wide array of exploratory outcome measures to measure neuroprotective effects.

The primary outcome measure was safety, which was met, indicating that the additional regiment of anti-rejection and antiviral treatments was successful. After 3 years, survival was 100%. There were no acute complications, with most only showing a low-grade transient headache and neck stiffness, which can be attributed to the lumbar puncture. In those individuals where cerebrospinal fluid (CSF) was available 3 months after treatment, analyses indicate the presence of donor cells, implying longer-term viability of these NPCs. These results clearly open the door for phase 2 and 3 trials.

Secondary outcomes of clinical activity and progression showed no effect, which is disappointing but perhaps require longer time windows. In fact, it is known that current standard clinical outcome measures are often insensitive to subtle changes in neurological functions and need a relatively long follow-up period to detect characteristic progressive deterioration, especially in PMS.^6^ It should also be noted that half of all affected individuals formed new lesions, which is unexpected in such a progressive population with already severe disability (EDSS 7). The presence of inflammatory activity is especially surprising with the concomitant use of tacrolimus to prevent rejection of transplanted NPCs. This concomitant immune activity could also have masked neuroprotective effects on all outcome measures and requires monitoring in subsequent larger studies, although there was no correlation with treatment dose in the current investigation.

It is important that cognitive assessments were also included, given their high correlations with atrophy.^2^ No effects were found, possibly due to the lack of a larger battery of tests and control data but also the presence of baseline differences between groups. Similar negative results were seen for plasma biomarkers, neurophysiological and ophthalmological measures known to be relevant for MS. Interestingly, all changes over time were not related to treatment dose, also implying that there was no worsening related to treatment, highlighting safety.

Perhaps most interestingly, advanced MRI analyses showed an effect for gray matter volume loss. Rates of this neurodegenerative change during a 2-year follow-up period were lower in individuals treated at higher doses compared with those at lower doses, with a trend for whole-brain volume loss. Both rates also directly correlated with the number of injected NPCs, even after correcting for baseline neurodegeneration and the formation of new lesions. This direct correlation thus implies a specific neuroprotective effect that warrants further study. This effect could be driven by repair mechanisms induced by the NPCs, as previous preclinical work in models of MS suggests specific migration to demyelinated areas inducing such repair.^7^ Whether this effect is also at play here remains difficult to conclude because of the limits of *in vivo* monitoring of such changes. Future studies could also incorporate regional measures of neurodegenerative change that have been shown to be especially powerful at explaining clinical heterogeneity and treatment response, such as thalamic volume.^8^

Finally, an impressive array of CSF analyses was performed, indicating an upregulation of trophic factors and immune-related molecules and cytokines/chemokines, although these could be driven by the additional immunomodulatory treatment and/or high immune activity of this cohort. More interestingly, an advanced proteomics analysis showed an enrichment of cellular pathways responding to growth factors and neuroplasticity. Such advanced pathways are highly interesting but remain difficult to implement in a clinical setting, which is more feasible for neurofilament light (NfL) and glial fibrillary acidic protein (GFAP),^9^ both of which unfortunately showed no positive treatment effect. However, this effect could be masked because of their additional relation with neuro-inflammatory changes.

Some unanswered questions remain. Firstly, it is unclear how long NPCs remain viable in a human setting and toward which cells these stem cells differentiate. Further exploring mechanisms of action would be of high interest given that earlier work has shown the presence of oligodendrocyte precursor cells in PMS, which appear inhibited in their maturation.^10^ Secondly, neurodegenerative effects are impressive but also warrant some caution, given the high inflammatory activity of the cohort, which impacts measurement due to edema, but could also impact possible neuroprotective effects induced by NPCs. In addition, the lack of clinical impact of the neuroprotective effect warrants longer follow-up with additional clinical biomarkers, as it remains unclear whether a purely neuroprotective effect is sufficient to achieve meaningful benefit for an affected individual. Finally, this study specifically chose individuals with relatively severe disease courses and failure of previous DMTs, while neuroprotective strategies might be especially beneficial to prevent such disease progression, possibly warranting future trials in earlier disease stages.

In summary, this important study is a first step toward NPC-based neuroprotection in MS, showing a feasible and safe approach. As neuroprotection is taking center stage in the treatment of people with MS, we eagerly await results from subsequent phase 2 and 3 trials in the future.

## References

[1] Genchi A., Brambilla E., Sangalli F., Radaelli M., Bacigaluppi M., Furlan R., Andolfo A., Drago D., Magagnotti C., Scotti G.M. (2023). Neural stem cell transplantation in patients with progressive multiple sclerosis: an open-label, phase 1 study. Nat. Med..

[2] Kuhlmann T., Moccia M., Coetzee T., Cohen J.A., Correale J., Graves J., Marrie R.A., Montalban X., Yong V.W., Thompson A.J. (2023). Multiple sclerosis progression: Time for a new mechanism-driven framework. Lancet Neurol..

[3] Burt R.K., Balabanov R., Burman J., Sharrack B., Snowden J.A., Oliveira M.C., Fagius J., Rose J., Nelson F., Barreira A.A. (2019). Effect of nonmyeloablative hematopoietic stem cell transplantation vs continued disease-modifying therapy on disease progression in patients with relapsing-remitting multiple sclerosis: A randomized clinical trial. JAMA.

[4] Tur C., Carbonell-Mirabent P., Cobo-Calvo A., Otero-Romero S., Arrambide G., Midaglia L., Castillo J., Vidal-Jordana A., Rodriguez-Acevedo B., Zabalza A. (2023). Association of early progression independent of relapse activity with long-term disability after a first demyelinating event in multiple sclerosis. JAMA Neurol..

[5] Cagol A., Schaedelin S., Barakovic M., Benkert P., Todea R.A., Rahmanzadeh R., Galbusera R., Lu P.J., Weigel M., Melie-Garcia L. (2022). Association of brain atrophy with disease progression independent of relapse activity in patients with relapsing multiple sclerosis. JAMA Neurol..

[6] Koch M.W., Mostert J., Repovic P., Bowen J.D., Comtois J., Strijbis E., Uitdehaag B., Cutter G. (2022). The timed 25-foot walk is a more sensitive outcome measure than the EDSS for PPMS trials: An analysis of the PROMISE clinical trial dataset. J. Neurol..

[7] Pluchino S., Quattrini A., Brambilla E., Gritti A., Salani G., Dina G., Galli R., Del Carro U., Amadio S., Bergami A. (2003). Injection of adult neurospheres induces recovery in a chronic model of multiple sclerosis. Nature.

[8] Filippi M., Preziosa P., Langdon D., Lassmann H., Paul F., Rovira A., Schoonheim M.M., Solari A., Stankoff B., Rocca M.A. (2020). Identifying progression in multiple sclerosis: New perspectives. Ann. Neurol..

[9] Magliozzi R., Cross A.H. (2020). Can CSF biomarkers predict future MS disease activity and severity?. Mult. Scler..

[10] Zveik O., Fainstein N., Rechtman A., Haham N., Ganz T., Lavon I., Brill L., Vaknin-Dembinsky A. (2022). Cerebrospinal fluid of progressive multiple sclerosis patients reduces differentiation and immune functions of oligodendrocyte progenitor cells. Glia.

